# Approach to Complementary Feeding and Infant Language Use: An Observational Study

**DOI:** 10.1111/mcn.13762

**Published:** 2024-11-11

**Authors:** Claire Farrow, Jacqueline Blissett, Shefu Islam, Rachel Batchelor, Rebecca Norman, Charlotte Webber, Elsa Addessi, Francesca Bellagamba, Amy T. Galloway, Laura Shapiro

**Affiliations:** ^1^ School of Psychology, Institute of Health and Neurodevelopment Aston University Birmingham UK; ^2^ Loughborough University Loughborough UK; ^3^ University of Oxford Oxford UK; ^4^ University College London London UK; ^5^ The University of Edinburgh Edinburgh UK; ^6^ Institute of Cognitive Sciences and Technologies National Research Council of Italy Rome Italy; ^7^ Sapienza University of Rome Rome Italy; ^8^ Appalachian State University Boone North Carolina USA

**Keywords:** complementary feeding, language development, mealtime observations, parenting

## Abstract

Emerging research suggests that a more infant‐led approach to complementary feeding may confer benefits for child language, but these findings are based on parent report studies. Using an observational approach this study examines whether different complementary feeding experiences relate to infant language exposure and language use. Fifty‐eight parents recorded a typical infant mealtime in the home (mean infant age = 14 months, SD = 4.15). Observations were coded to measure the prevalence of infant‐led and parent‐led feeding using the Family Mealtime Coding Scheme. Caregiver language use (word types and token directed at the child, mean length of utterances in child‐directed speech, responsiveness and initiations) and the number of infant vocalisations were coded in ELAN using CHAT conventions and parents completed the MacArthur Communicative Development Inventory short form as a measure of child language. Greater observed infant self‐feeding was significantly associated with greater observed exposure to language from caregivers (*r* = 0.312 percentage of infant self‐feeding correlated with caregiver word types directed at the child) and a greater number of infant vocalisations (*r* = 0.320 percentage of infant self‐feeding correlated with number of child vocalisations produced). Structural Equation Modelling showed the relationship between infant self‐feeding and infant vocalisations to be significantly mediated by enhanced quality and quantity of caregiver child‐directed speech (model fit: *χ* ^2^ [5] = 5.01, *p* = 0.415, CFI = 1.00 [NF = 0.98], RMSEA = 0.006). Differences in the approach to complementary feeding may shape infant's experiences in ways that support language exposure and use. Autonomy associated with infant self‐feeding may enhance opportunities for social interaction.

## Summary

- The complementary feeding period has lifelong consequences for health and well‐being. A more infant‐led approach to feeding which offers the child more independence to feed themselves has been associated with positive eating behaviour outcomes for children.
- During home‐based mealtime observations, infants who fed themselves more often also produce more vocalisations at mealtimes. These infants were also more likely to be exposed to language from their caregivers.
- The relationship between infant self‐feeding and infant vocalisations was explained by the greater quality and quantity of caregiver speech directed at the infant.
- Greater infant autonomy associated with infant self‐feeding may enhance opportunities for social interaction.
- The different ways that infants are offered food during the complementary feeding period can shape infant's experiences in ways that may support their language exposure and language use.

## Graphical abstract

Infants who fed themselves more often during mealtime were observed to produce more vocalisations. Greater infant self‐feeding was associated with greater caregiver language use, and the quality and quantity of caregiver speech explained the relationship between greater infant self‐feeding and greater infant vocalisations.

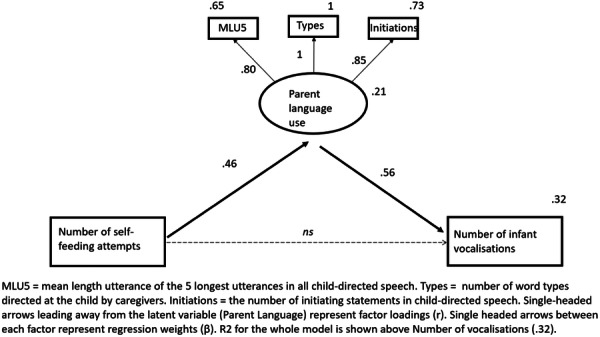

**Abbreviations**
AMOS: analysis of a moment structures
CFI: comparative fit index
CHAT: Child Language Data Exchange System
CLAN: computerised language analysis
ELAN: EUDICO Linguistic Annotator
MLU5: mean length of the 5 longest utterances in all child‐directed speech
RMSEA: root mean square error of approximation
SD: standard deviation.

## 1 Introduction

A considerable amount of research has been conducted to understand how the approach to milk‐feeding affects child development (Victora et al. 2016), but there has been a lack of research exploring the consequences of infant's transitioning experiences to solid foods during the stage of complementary feeding (Sachs 2011). Recent years have seen increased attention given to the greater prevalence of infant‐led complementary feeding, often popularised as ‘baby‐led’ weaning (Brown, Jones, and Rowan 2017), but there has been little exploration of the relationship between approach to complementary feeding and developmental outcomes. The first randomised controlled trial in this area focussed on child eating behaviour found that a modified infant‐led approach (which also encouraged intake of iron‐rich foods) was associated with lower child food‐fussiness, greater enjoyment of food (Taylor et al. 2017) and a diet as nutritionally adequate as spoon feeding (Erickson et al. 2018). Experiences during complementary feeding may also shape development more widely for children. For example, an infant‐led approach is associated with greater participation in family mealtimes (Brown and Lee 2010) which offers increased exposure to language from multiple caregivers and other children (Beals 1997; Snow and Beals 2006). The average infant spends 11.8 h per week eating at home (Hofferth and Sandberg 2001), and eating together with other family members offers opportunities for social interaction and modelling joint interaction, which can support linguistic development (Snow and Beals 1997). Eating solid foods, compared to being fed puréed foods, also requires the infant to practice more complex oral‐motor and fine‐motor movements (Cichero 2016), which may enhance the skills needed for language development (Alcock 2006; Gernsbacher et al. 2008; LeBarton and Iverson 2013). Increased chewing, biting and mastication associated with consuming a more solid diet may promote craniofacial growth and the strengthening of facial muscles which are important for the development of speech (Abed et al. 2007).

In the first study to explore potential relationships with language, Webber et al. (2021) found positive relationships between a more infant‐led approach to feeding and language outcomes, a relationship mediated by how often parents said that the child ate family meals. However, this study relied on parental report, which may be subject to bias, and further observational and experimental work is needed. The aim of the current study is to explore whether different mealtime experiences during complementary feeding relate to differences in language exposure and language use at mealtimes. We use an observational approach to study naturally occurring experiences within the home. Caregivers report that they often blend parent‐led and infant‐led approaches to feeding (with 44% combining elements of each; Komninou, Halford, and Harrold 2019), so we assess how often infants self‐feed and do not seek to compare groups according to approach to weaning. It is hypothesised that a greater prevalence of behaviours indicative of infant‐led feeding will be associated with caregiver and infant language use and that observed exposure to adult language at mealtimes will mediate any relationship between infant self‐feeding and infant language.

## 2 Method

### 2.1 Participants

Fifty‐eight caregivers completed home observations of infant mealtimes, and 51 also completed questionnaire measures. Participants included 37 mothers and 14 fathers (mean age = 30.55 years, SD = 4.12).

### 2.2 Procedure

Participants were invited to participate using the online recruitment platform ‘Prolific’ if they had registered as parents of children under 2 years old. The study was advertised for parents who had commenced complementary feeding. Participants completed a background questionnaire, including questions about complementary feeding experiences. They were invited to record a typical mealtime for their infant. About 98% of people aged < 35 have access to smartphones with sophisticated recording devices (Statistica 2021); participants were therefore invited to use a device such as a mobile phone/recording camera to record mealtimes but advised that a device would be provided if needed (no participants requested this). Participants were asked to record observations at home to allow them to participate with minimal interference and to reduce bias resulting from a researcher being present. They were given instructions to maximise the quality of recordings. Ethical approval for the study was given by Aston University. All participants were provided with an information sheet about the study and provided written informed consent for themselves and their child to take part.

#### 2.2.1 Measures

Parents completed a background questionnaire about their relationship to the child, their age, ethnicity, education, child age and gender. Parents also completed a language questionnaire about their child's language comprehension and production. They also completed a complementary feeding experiences questionnaire asking about the age of introducing foods other than milk and the approach to complementary feeding used (whether the infant was parent‐fed or self‐fed and whether puréed/mashed/finger foods were used). If the infant was weaned using puréed or mashed foods, parents were asked at what age the infant started eating finger foods and family foods. Parents were also asked about current feeding practices, specifically: how often the child is offered puréed food, how often the child is spoon‐ or fork‐fed by an adult and how often the child eats with the rest of the family. Response options ranged from 0% to 100% of the time. Questions were adapted from previous infant feeding questionnaires (Brown and Lee 2010; Cameron, Heath, and Taylor 2012).

##### 2.2.1.1 Child Language Questionnaire

Parents completed the MacArthur Communicative Development Inventory (CDI short form; Fenson et al. 2000) as a measure of child language comprehension and production. The MacArthur Inventories are widely used to assess language development, and the short forms show good reliability and validity from 8 months (Fenson et al. 2000). Parents are asked to select which words the child comprehends and produces from an 89‐word vocabulary list (Fenson et al. 2000). Percentile scores are computed standardised for child gender and age in months. Although our main measures of language are derived from the mealtime observations detailed below, this general measure of language was used to provide normative data for the sample.

##### 2.2.1.2 Observations of Mealtime Interactions

The Family Mealtime Coding Scheme (Haycraft and Blissett 2008) was used to code mealtime interactions. The original measure was developed for children > 18 months old; therefore, some codes were adapted for suitability with younger children. Background codes included where the child was sitting (e.g., highchair, floor), the number of adults and children present, whether distractions (e.g., tablet, TV, toys) were used at the mealtime and meal length. Counts were made for the number of times the child fed themselves, including successful and unsuccessful attempts to feed with fingers, utensils or parent support (e.g., parent pre‐loading a spoon of food and assisting the child to feed). The number of times an adult fed the child using their fingers or utensils was also coded. Codes were taken from the point food was placed in front of the child and were computed for the whole mealtime to capture the range and quantity of variation in feeding and eating experiences. Counts for infant self‐feeding and parent‐feeding were also computed for up to 20 min or the first 20 min only to account for variability arising from longer mealtimes. Mealtime behaviours were coded by two trained researchers, SI and RB, with support and training from CF using the Behavioural Observation Research Interactive Software (Friard and Gamba 2016). Twenty‐two (38%) observations were double coded to assess two‐way random inter‐rater reliability, which was high at 0.99 (*p* < 0.001) for all feeding measures.

##### 2.2.1.3 Observations of Mealtime Language Use

Mealtime language use by parents and children was coded. We used a multi‐media annotation tool called ELAN (Sloetjes and Wittenburg 2008) to enter, display and work with the transcripts. We used CHAT‐Transcription conventions as our transcription format and CLAN to analyse the transcribed language data (MacWhinney 2008). Caregiver speech codes included the number of word types directed at the child, the number of word tokens directed at the child, the mean length of utterance in words for all child‐directed speech, the mean length of the five longest utterances in all child‐directed speech (MLU5), the number of utterances that were responsive to the child and the number of initiating statements in child‐directed speech. The number of vocalisations produced by infants was coded. Transcriptions of language were made by a trained researcher, RN, not involved with mealtime behaviour coding. About 28% of transcriptions were second coded by a trained researcher and discrepancies were resolved with LS. Coding was computed for the first 20 min only to standardise for observation length. Inter‐rater reliability was high with interclass correlations at 1 (*p* < 0.01) for all counts of child‐directed speech.

### 2.3 Results

Descriptive statistics were computed to characterise the sample. Pearson's two‐tailed correlations were used to explore whether behaviours indicative of infant‐led feeding were associated with caregiver and child language use. Structural equation modelling using AMOS assessed whether observed exposure to adult language at mealtimes mediated a relationship between observed infant self‐feeding behaviour and observed child language use.

#### 2.3.1 Participant Characteristics and Descriptive Statistics

Participant characteristics are described in Table 1. The mean infant age was 14 months.

**Table 1:** Participants characteristics.

	Mean (SD), Group N or %
Parent age (years)	30.55 (4.12)
Parent sex	37 mothers
	14 fathers
Mean education post‐16 (years)	5.18 (2.16)
Parent ethnicity (%)	92% White
	4% Black
	2% Indian
	23% Dual‐heritage
Infant age (months)	14 (4.15)
Infant sex	32 boys
	19 girls

#### 2.3.2 Descriptive Statistics

According to parental report, the mean age of introducing food other than milk was 5.63 months. When infants started complementary feeding, the modal approach to feeding was for the infant to sometimes feed themselves unaided (37%) and parents to often feed the infant puréed or mashed foods rather than finger foods (33%). Many children started eating finger foods when complementary feeding started (40%) and many ate family foods 2–4 months after complementary feeding began (43%). For current feeding practices, caregivers reported that children were offered puréed food on average 17% of the time and were spoon‐ or fork‐fed by an adult 38.4% of the time. Caregivers reported that children ate with the rest of the family on average 80.2% of the time.

For mealtime observations, most families fed their children using a high chair or at a table (94.8%) with 1.7% sitting on the floor and 3.4% sitting on their caregiver's lap. For 67.2% of observations there was one adult present during the mealtime, two adults present in 29.3% of observations and three adults present in 3.5% of observations. In 96.6% of observations, there were no other children present. About 44.8% of children had a distraction (e.g., electronic tablet, television) audible to them during mealtimes. Mean counts for feeding, eating and language codes are presented in Table 2. On average, children fed themselves 33 times and caregivers fed children 12 times. The average percentage of self‐feeding as a ratio of overall eating mouthfuls was 71% (with range = 0%–100%, SD = 34.74). During the first 20 min of mealtime observations, infants fed themselves 29 times on average and caregivers fed infants nine times on average. The average speed of eating (computed as the total number of mouthfuls from parent or child/meal length in seconds * 60) was 2.99 mouthfuls per minute.

**Table 2:** Descriptive statistics for measures of feeding and language use.

	Mean (SD)	Range
Parent‐reported child language use		
MacArthur CDI percentile score—language production	30.56 (31.80)	5–99
MacArthur CDI percentile score—language comprehension	45.98 (30.21)	5–99
Observations of mealtime behaviour:		
• Child self‐feeding mouthfuls (for total meal) (*N*)	33.67 (32.97)	0–203
• Caregiver finger feeding mouthfuls (for total meal) (*N*)	2.67 (7.46)	0–42
• Caregiver feeding with utensils mouthfuls (for total meal) (*N*)	9.5 (13.70)	0–63
• Total number of feeding instances (parent and child combined) (for total meal) (*N*)	50.34 (34.06)	0–215
• Speed of eating^a^	2.99 (1.35)	0–6.76
• Percentage of self‐feeding overall during mealtime (for total meal) (%)	71.96 (34.74)	0–100
• Number of instances of self‐feeding (during first 20 min of mealtime only)	28.57 (26.41)	0–140
• Number of instances of caregiver feeding (during first 20 min of mealtime only)	8.66 (13.78)	0–70
Observations of mealtime language use (during first 20 min of mealtime):		
• Word types directed at child (*N*)	154.71 (82.21)	12–425
• Word tokens directed at child (*N*)	548.07 (446.20)	24–2101
• Mean length of utterance in child‐directed speech	3.48 (0.86)	1.79–5.65
• Mean length of 5 longest utterances in child‐directed speech	10.85 (4.00)	3–22.20
• Language responses in child‐directed speech	11.9 (13.9)	0–62
• Language initiations in child‐directed speech (*N*)	137.03 (94.31)	10–445
• Child vocalisations produced (*N*)	78.86 (50.36)	10–195

^a^ Speed of eating as rate per minute = (total number of mouthfuls/meal length in seconds) × 60.

Table 2 shows parent‐reported child language use as age‐adjusted percentile scores. Overall, scores for our sample were lower than age‐matched norms for language production (31st percentile) although overall scores for language comprehension are close to expectations (46th percentile). In terms of mealtime language use, in the first 20 min of observations, caregivers uttered an average of 155 word types and 548 word tokens directed at the child, and they initiated speech 137 times and responded 11 times. The mean length of utterance directed at the child was three words and the mean length of the five longest utterances (MLU5) was 11 words. On average, children produced 79 vocalisations of any sort during mealtime (including words, proto‐words and meaningful sounds, e.g., ‘brum brum’).

#### 2.3.3 Observations of Complementary Feeding Behaviour and Language Use

Parental education and age were not correlated with parental language use during mealtime observations, or with child age, or the percentage of self‐feeding. There were no significant differences (using *t*‐tests) in feeding or language use measures according to child sex. Child age was correlated with feeding variables but not with mealtime language use, suggesting that child age was not a key factor in explaining individual differences in caregivers’ mealtime language. Child age was also not correlated with number of child vocalisations. Note that this is a measure of language quantity (not quality) and a vocal young child may well elicit as many vocalisations as an older child speaking in sentences. Our parent‐reported language measures showed an expected increase in language comprehension and production with age before conversion to age‐adjusted percentiles. In addition, parent‐reported language measures were correlated with observed language measures. The number of parental responses to child vocalisations observed during mealtimes was significantly and positively correlated with parent‐reported child comprehension percentile (*r* = 0.488, *p* < 0.01) and parent‐reported child production percentile (*r* = 0.537, *p* < 0.01). There were no other significant correlations between parent‐reported child language and observed child language. The percentage of child self‐feeding was positively correlated with the number of word types directed at the child, mean length utterance in child‐directed speech, MLU5 and the number of child vocalisations produced (see Table 3).

**Table 3:** Relationships between observed parent‐ and infant‐led feeding behaviours and observed language use at mealtimes.

	0	1	2	3	4	5	6	7	8	9	10
0. Parent education											
1. Child age	−0.063	—									
2. Percentage of child self‐feeding entire meal	−0.263	0.341*	—								
3. Counts of infant self‐feeding (0–20 min)	−0.224	−0.106*	—								
4. Counts of parent feeding (0–20 min)	0.183	−0.296*	−0.729**	−0.355**							
5. Caregiver word types directed at child (0–20 min)	0.159	−0.010	0.312*	0.438**	−0.025	—					
6. Caregiver word tokens directed at child (0–20 min)	0.218	−0.088	0.212	0.387**	0.050	0.941**	—				
7. Mean length utterance in child‐directed speech (0–20 min)	−0.131	0.108	0.458**	0.373**	−0.214	0.637**	0.576**	—			
8. Mean length of five longest utterances in child‐directed speech (0–20 min)	0.006	0.046	0.403**	0.301*	−0.105	0.833**	0.779**	0.803**	—		
9. Language responses in child‐directed speech (0–20 min)	0.149	0.212	0.213	0.104	−0.078	0.433**	0.375**	0.115	0.208*	—	
10. Language initiations in child‐directed speech (0–20 min)	0.262	−0.133	0.092	0.274*	0.193	0.881**	0.941**	0.364**	0.683**	0.320**	—
11. Observed number of child vocalisations produced (0–20 min)	−0.103	0.084	0.320*	0.281*	−0.161	0.609**	0.583**	0.354**	0.523**	0.570**	0.540**

*Note:* Pearson's two‐tailed correlations (*N* = 58).

* *p* < 0.05

** *p* < 0.01.

#### 2.3.4 Testing the Mediation Model

The pattern of correlations in Table 3 was used to determine measures for SEM. Given the negative correlation between child self‐feeding and the prevalence of parent feeding (reflecting the fact that the more frequently a child attempts to self‐feed, the less frequently the parent needs to feed them), we chose not to use parental feeding as a measure in our SEM models. We coded two measures of mean length utterance in child‐directed speech and found that the MLU5 correlates more strongly with other parent‐language measures and therefore included this measure. Finally, both parent language initiations and responses were significantly correlated with child vocalisations. However, because responses are dependent on the nature of child vocalisations, we included only language initiations as a more independent measure of language exposure.

**Figure 1:**
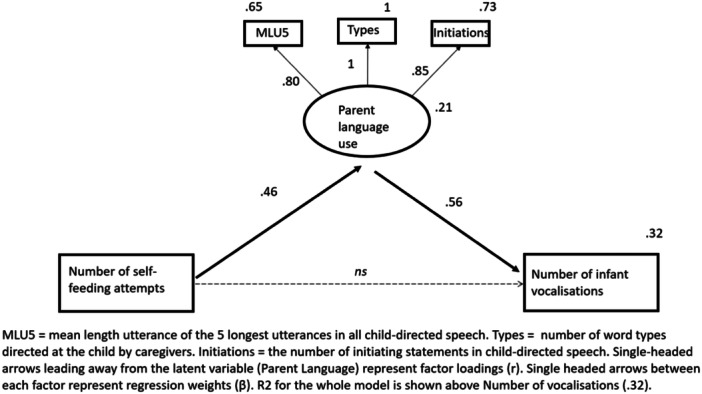
Structural equation model of the relationship between infant self‐feeding and the number of infant vocalisations at mealtimes, mediated by parental language use.

We used SEM to test our hypothesis that the amount of child self‐feeding predicts the number of child vocalisations during feeding, mediated by parent language (specifically the quality and quantity of child‐directed speech). See supplementary material for further explanation for model choices. The full mediation model is shown in Figure 1. Full information maximum likelihood was used to handle missing data. Models were compared using Chi‐square tests. The goodness‐of‐fit of the models was evaluated with the Chi‐square statistic (*χ* ^2^), the comparative fit index (CFI) and root mean square error of approximation (RMSEA). The criteria for a well‐fitting model were: a nonsignificant *χ* ^2^ value, CFI > 0.96 and the RMSEA < 0.06 (Hu and Bentler 1999). Raw scores were used for all measures. As shown in the model below, the regression weights are indicating that self‐feeding significantly predicts parent language, which significantly predicts child vocalisations: model fit: *χ* ^2^ (5) = 5.01, *p* = 0.415, CFI = 1.00 (NFI = 0.98), RMSEA = 0.006, 90% CI = 0.000–0.184. When the direct link between child self‐feeding and child vocalisations was included, the regression weight was not significant: Beta = 0.06 (standardised Beta = 0.03), *p* = 0.804, and the fit indices were not significantly improved (delta *χ* ^2^ = 0.06), leading us to accept the full mediation model as the best fit.

### 2.4 Ethics Statement

Ethical approval for the study was provided by Aston University Research Ethics Committee.

## 3 Discussion

Advanced child language development predicts better outcomes in a range of different domains (Zauche et al. 2017; Hebert‐Myers et al. 2006). This study is the first to find that observations of greater infant‐led feeding behaviours during complementary feeding are positively associated with observations of greater child language use and that this relationship is mediated by the quantity and quality of caregiver language. These findings highlight the valuable role that mealtimes offer for enhancing infant's language exposure and use and shed light on the opportunities offered by the formative period of complementary feeding when caregivers and infants tend to be together during mealtimes and occasions for hearing caregiver vocalisations may be particularly enhanced.

Our SEM model emphasises that the relationship between child self‐feeding and vocalisations is explained by caregiver language use during mealtimes. It is already established that adult language exposure is causally related to children's language development (Topping, Dekhinet, and Zeedyk 2013). Previous research suggests that variation in parental language use can be explained by broad factors such as culture, SES and ethnicity, but positive associations between language exposure and language development are seen across all demographic groups (Anderson et al. 2021), and there remains variation across language exposure that is unrelated to these contextual differences (Hoff 2006). Here, we find that the complementary mealtime experience is one place where this variance occurs: child vocalisations were correlated with the quantity of caregiver word tokens and types, with initiations and responsiveness in child‐directed speech, as well as the quality of utterances in terms of complexity.

Although statistically the SEM model could imply a causal pathway, these findings are based on cross‐sectional data which may not account for other potential predictors of language use not captured in this study. The fact that self‐feeding appears to predict greater parental language use might suggest that providing the child with increased autonomy at mealtimes reduces the task‐focussed nature of infant feeding and allows mealtimes to be more dyadic, interactive and social in nature. This suggestion requires testing with an experimental design to better understand what is driving differences in parental language use at mealtimes and whether these are reflective of a change in behaviour or resulting from other confounding variables related to parenting characteristics, differences between children (e.g., child development and food fussiness may shape eating behaviour and language use), or other social or demographic confounds.

Aside from the exposure to adult language during feeding interactions, there are multiple potential other ways in which child self‐feeding may enhance language exposure. Infants who are self‐feeding more often are likely consuming a more solid diet which requires the use of more complex oral‐motor functions that are positively correlated with language outcomes (Alcock 2006). Moreover, the act of self‐feeding requires repeatedly practising fine motor movements to grasp food alongside hand‐eye coordination to bring food to the mouth. More infant‐led feeding has been associated with unsupported sitting and earlier crawling (Addessi et al. 2021) as well as marginally higher grasping skill score and fine motor quotient (Campeau et al. 2021). Infant advances in motor development may allow them to expand interactions with the environment in ways that benefit language; for example, unsupported sitting has been shown to free the rib cage, allowing more deep breathing and maintenance of a subglottal pressure that allows infants to produce longer strings of utterances and consonant‐vowel segments (Yingling 1981). Future research is needed to understand whether potential advantages in terms of oral and fine‐motor experiences afforded by self‐feeding may offer advantages for language development.

There has been debate about the benefits and limitations of spoon‐feeding infants beyond 6 months, with some suggestions that spoon‐feeding may hinder oral motor skills and satiety responsiveness (Rapley 2016). In this study, we only explore associations with language use but we did not find that parental feeding was negatively associated with parental language use or with child vocalisations, suggesting that it is the act of self‐feeding that may be driving a relationship with language exposure and use rather than the absence of parental feeding per se. As part of this study, we chose to measure naturally occurring behaviours in the child's home; we did not compare groups of caregivers who said that they did or did not follow a particular weaning plan. This is important because parents who follow a strict infant‐led approach to feeding generally differ from other parents in terms of education, occupation, personality and parenting style (Brown and Lee 2010, 2011; Beals 1997). Our approach to not categorise families likely reflects experiences in the home where 44% of parents report that they use a mixture of puréed and infant‐led feeding (Komninou, Halford, and Harrold 2019), suggesting that they cannot be easily classified into groups according to complementary feeding approach.

The home‐based observational nature of the present study is a strength which offers a natural setting, particularly given that a researcher was not required to visit the home. We utilised detailed coding schemes of mealtime interactions and language utterances and independent coders to classify behaviour with excellent inter‐rater reliability. Limitations include the relatively small cross‐sectional study, above‐average parental education level, potential for observation bias and the homogenous ethnicity of the sample. A randomised controlled trial would advance understanding in this area and it would be interesting to understand whether relationships with parental language are specific to mealtimes or result in other general interactions. Further research is required to understand other potential mediators or moderators of the relationship between feeding approach and language outcomes, and it would be novel to explore whether there are differences between maternal and paternal interactions with infants. Given that the effect of parental language exposure on child language development appears to accumulate over time (Anderson et al. 2021), longitudinal studies are also required to explore whether this is indeed the case.

## 4 Conclusions

Infants who self‐fed more often at mealtimes also produced more language, and this relationship was explained by enhanced quality and quantity of caregiver child‐directed speech. The average infant spends substantial time eating at home each week (Hofferth and Sandberg 2001) and while the focus of those mealtime interactions has understandably been around healthy feeding and eating, this interactive time also offers opportunities for social interaction that may benefit infants in multiple other ways. Work with older children has already shed light on the surprising benefits of family mealtimes for avoidance of high‐risk behaviours and healthy development (Fruh et al. 2011). Thus, it seems that the potential range of benefits of shared eating interactions with infants and young children are only just beginning to be understood.

## Author Contributions

C.F., J.B., E.A., F.B., A.T.G. and L.S. designed the study. C.W. and C.F. collected the data. S.I., R.B. and R.N. coded the data. C.F. and L.S. led on analysis with feedback from all authors. All authors contributed to the review and editing of the paper, with C.F. as the lead author.

## Conflicts of Interest

The authors declare no conflicts of interest.

## Supporting information

Supporting information.

## Data Availability

The data that support the findings of this study are available on request from the corresponding author. The data are not publicly available due to privacy or ethical restrictions. Anonymised data are available from the first author on request. The analytic code necessary to reproduce the analyses presented in this paper is publicly accessible. The materials necessary to attempt to replicate the findings presented here are available from the first author. The analyses presented here were not preregistered.
