# Supplementary material for: Approach to Complementary Feeding and Infant Language Use: An Observational Study
Source: Matern Child Nutr. 2024 Nov 11;21(1):e13762. doi: 10.1111/mcn.13762 (PMC11650041; doi:10.1111/mcn.13762)
Supplement: Supplementary file 1 — Supporting information. [file MCN-21-e13762-s001.docx]

Supplementary material

Justification for SEM variable choices supplementary material

All measures utilised focussed on the first 20 minutes of the mealtime to standardise for the impact of longer meals. Measures of parent language were significantly correlated and allowed a latent variable for parent language to be included in the model. This latent variable included indicators of quantity and quality of child-directed speech (number of types, number of initiations and MLU5). The following indicators were not included as they were derived from the same measures: number of tokens (types was considered a better measure since it also taps into both quality and quantity), number of responses (dependent upon child vocalisations) and mean length of utterances (already captured in MLU5). Although we had three measures of feeding approach, only one measure tapped into the child’s feeding attempts and so we included an observed variable for child self-feeding. We also included a single observed variable for child vocalisations, to capture the full range of language production stages.
